# Supplementary material for: Cell culture NAIL-MS allows insight into human tRNA and rRNA modification dynamics in vivo
Source: Nat Commun. 2021 Jan 15;12:389. doi: 10.1038/s41467-020-20576-4 (PMC7810713; doi:10.1038/s41467-020-20576-4)
Supplement: Supplementary file 1 — Supplementary Information [file 41467_2020_20576_MOESM1_ESM.pdf]

Supplementary Information

Cell culture NAIL-MS allows insight into human tRNA and rRNA modification dynamics *in vivo*

Matthias Heiss[1], Felix Hagelskamp[1], Virginie Marchand[2], Yuri Motorin[2] and Stefanie Kellner[1,3]*

[1] Department of Chemistry, Ludwig-Maximilians-University Munich, Butenandtstr. 5-13, 81377 Munich, Germany

[2] Université de Lorraine, CNRS, Inserm, UMS2008/US40 IBSLor and UMR7365 IMoPA, F-54000 Nancy, France

[3] Institute of Pharmaceutical Chemistry, Goethe-University Frankfurt, Max-von-Laue-Str, 9, 60438 Frankfurt, Germany

Tel: +49-(0)89-218077724

Email: stefanie.kellner@cup.uni-muenchen.de and/or Kellner@pharmchem.uni-frankfurt.de

* To whom correspondence should be addressed

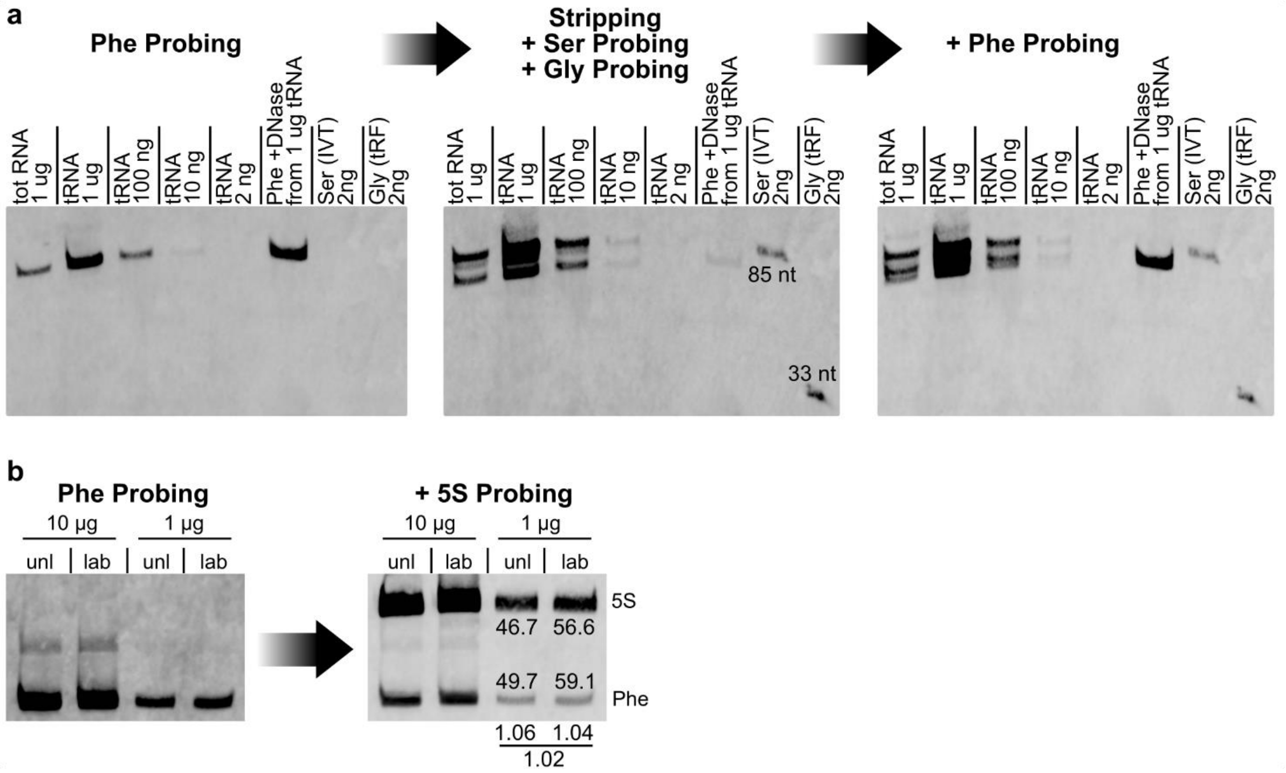

Figure S1: Northern Blot Analysis of purified tRNA^{Phe}. a Total RNA, total tRNA (1 µg to 2 ng dilutions) and tRNA^{Phe} (purified from 1µg total tRNA) were analyzed by urea PAGE (12 %) and visualized by northern blotting. Left: The abundance of tRNA^{Phe} in 1 µg total tRNA corresponds to the abundance found for tRNA^{Phe} isolated from 1 µg total tRNA, which indicates a high yield of tRNA^{Phe} preparation. Probing against tRNA^{Ser} and tRNA^{Gly} indicates a minor contamination of the purified tRNA^{Phe} with other tRNAs or unspecific binding of the probes. Subsequent probing against tRNA^{Phe} again ensures integrity of tRNA^{Phe} after stripping. The experiment was performed in a similar approach 3 times with high reproducibility of the results. **b** Total RNA (10 and 1 µg) from labeled (lab) and unlabeled (unl) cells was loaded on a 12 % urea PAGE and visualized by northern blotting. Left: hybridization with probe against tRNA^{Phe}. Right: subsequent hybridization of same membrane with probe against 5S rRNA as a loading control. ImageJ® was used to quantify band intensities (numbers in Blot). The abundance of tRNA^{Phe} is not influenced by the labeling procedure. This experiment was performed in a similar approach once and yielded comparable results.

Sequencing results for total tRNA

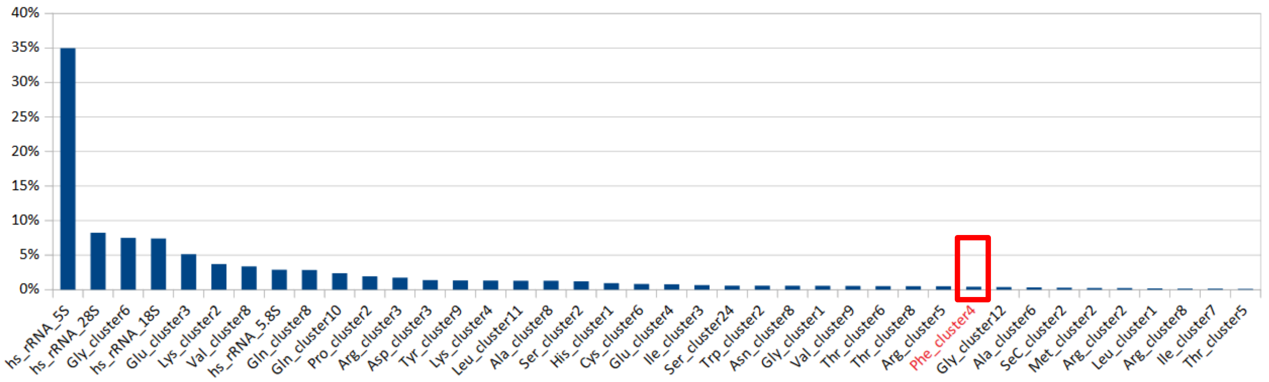

Sequencing results for $tRNA^{Phe}$

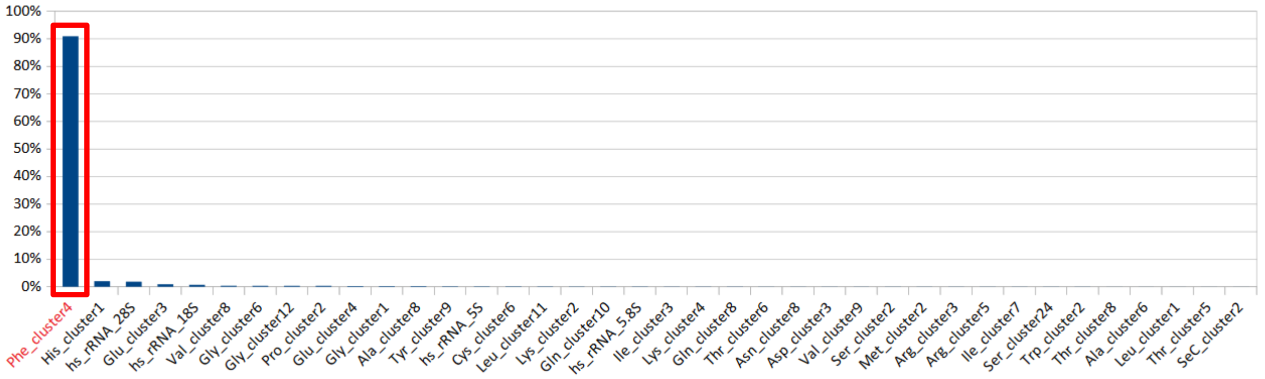

Figure S2: Sequencing of purified $tRNA^{Phe}$. Total tRNA, purified by size exclusion chromatography (top), and tRNAPhe, purified by oligonucleotide hybridization assay (bottom), were analyzed by conversion to libraries and sequencing. Alignment was performed to human rRNA and tRNA reference. $tRNA^{Phe}_{GAA}$ reads are boxed red.

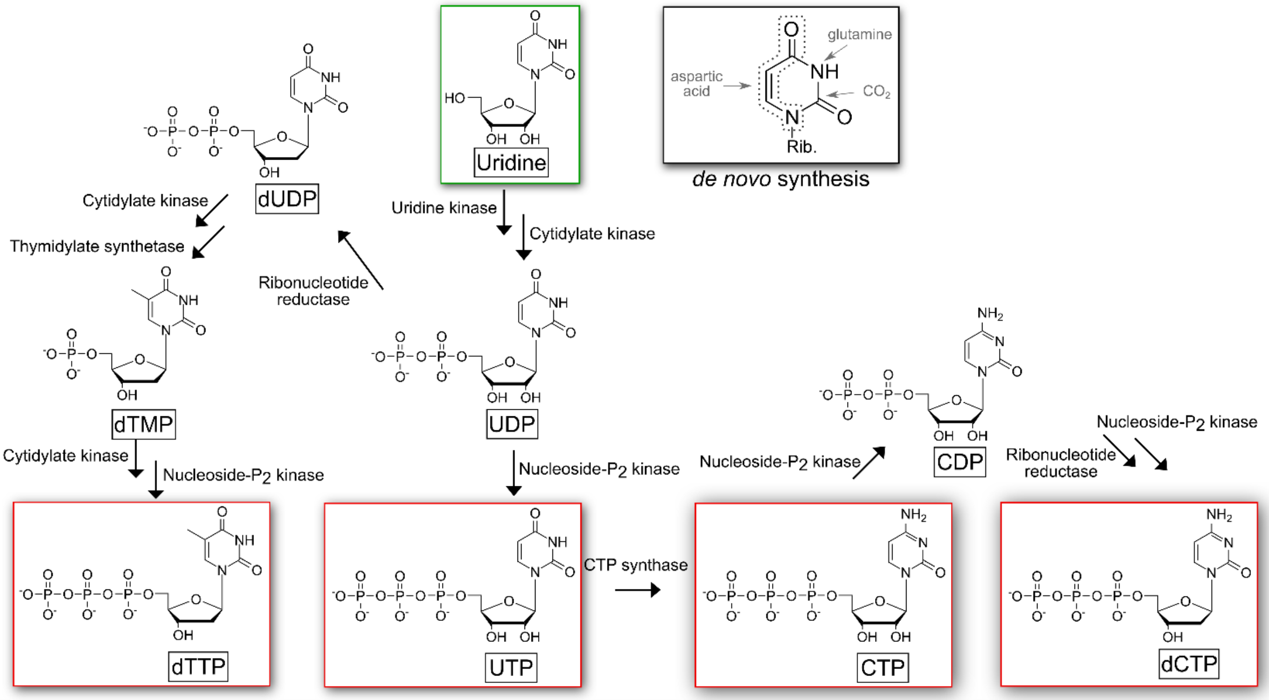

Figure S3A: Biosynthesis and connection of pyrimidines.
Uridine (unlabeled or labeled, green box) is taken up by the cell and then enzymatically converted to the canonical pyrimidine nucleotides for RNA and DNA biosynthesis (red boxes).

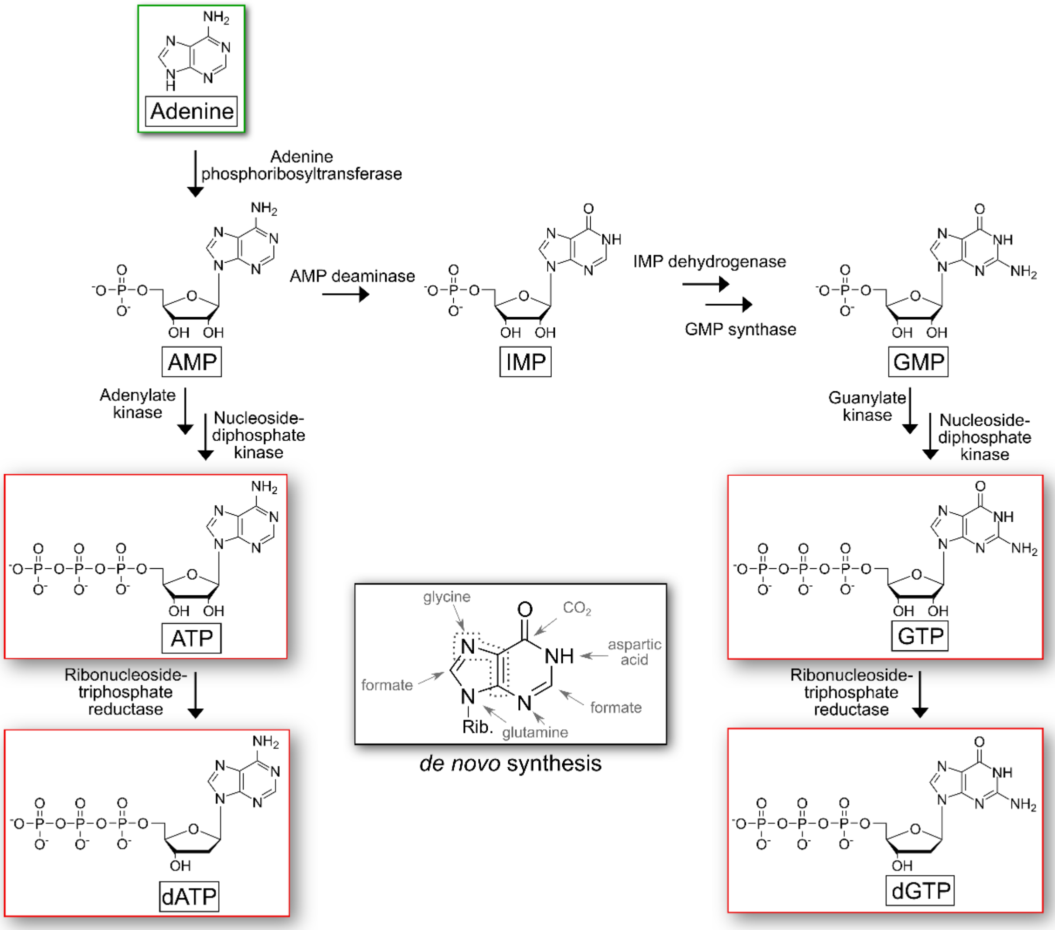

Figure S3B: Biosynthesis and connection of purines.
Adenine (unlabeled or labeled, green box) is taken up by the cell and then enzymatically converted to the canonical purine nucleotides for RNA and DNA biosynthesis (red boxes).

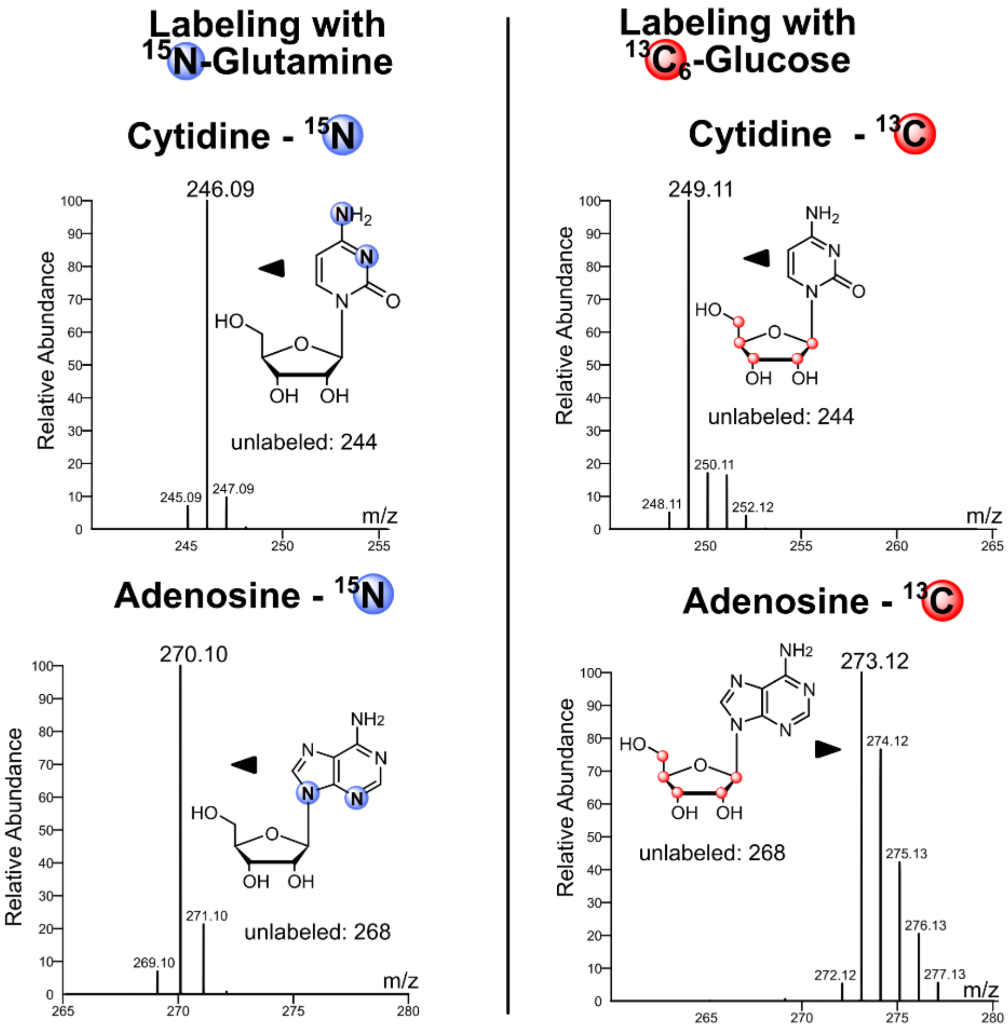

Figure S3C: Labeling of nucleosides with $^{15}N_2$-glutamine and $^{13}C_6$-glucose.
Cell culture medium was either supplemented with $^{15}N_2$-glutamine (left) or with $^{13}C_6$-glucose (right). Shown are the high-resolution mass spectra of cytidine (top) and adenosine (bottom) from isolated RNA after labeling for 7 days (=3 passages).

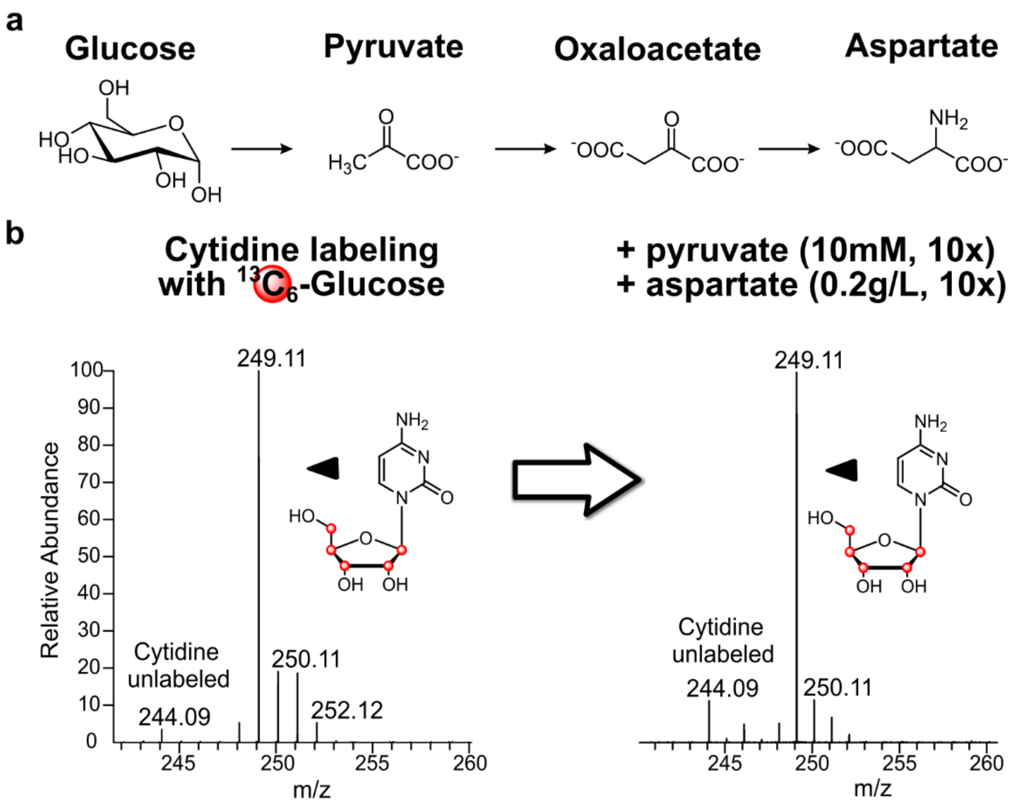

Figure S4: Pyrimidine labeling in $^{13}C_6$-glucose medium supplemented with pyruvate and aspartate.

a Reaction scheme showing the biosynthesis pathway of aspartate with pyruvate as an intermediate. **b** Left: High-resolution mass spectrum of cytidine after labeling with $^{13}C_6$-glucose. Right: High-resolution mass spectrum of cytidine with additional supplementation of pyruvate and aspartate to overcome poly-isotopic labeling. All cells were labeled for 7 days (=3 passages) in the respective medium.

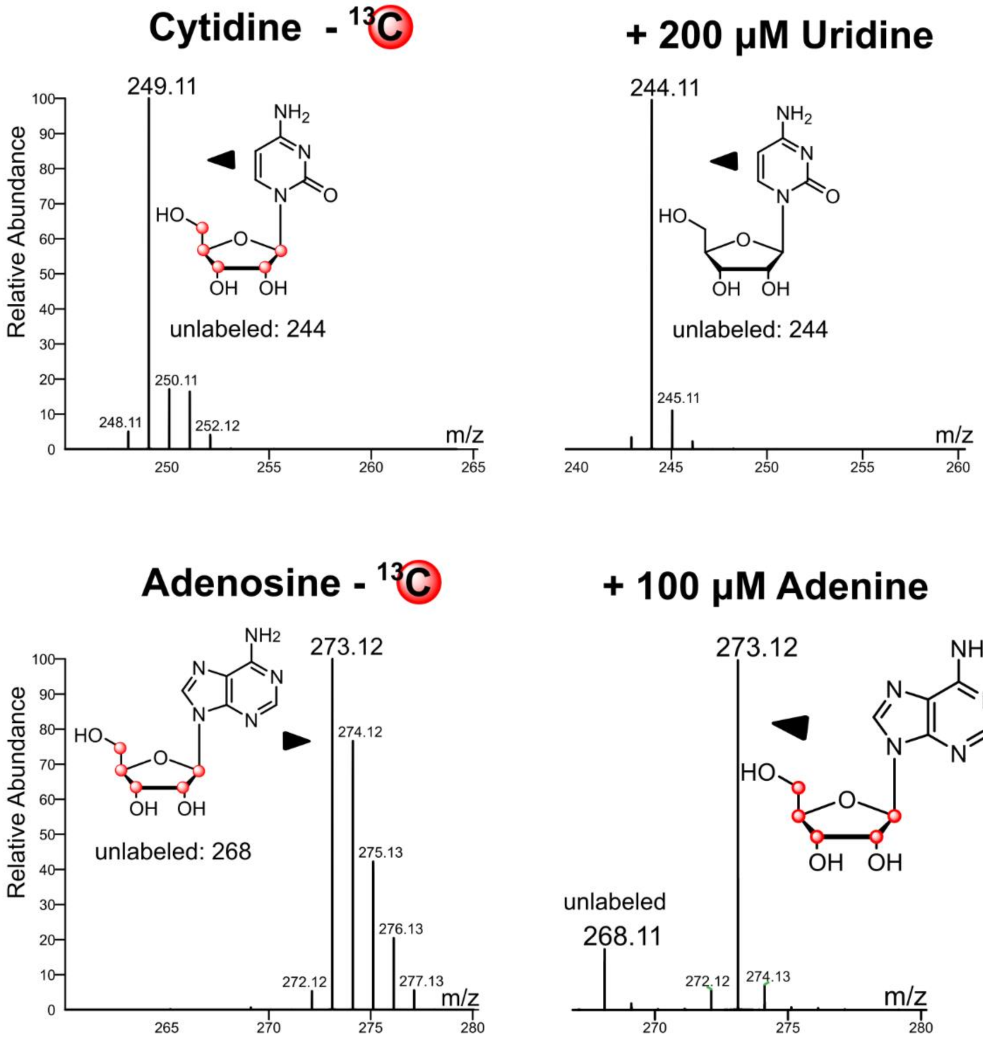

Figure S5: Improvement of nucleoside labeling in $^{13}C_6$-glucose medium by supplementation of uridine and adenine.

The high-resolution mass spectra on the left show labeling of cytidine (top) and adenosine (bottom) after labeling with $^{13}C_6$-glucose. The spectra on the right show the signals after additional supplementation with 200 µM uridine and 100 µM adenine. All cells were labeled for 7 days (=3 passages) in the respective medium.

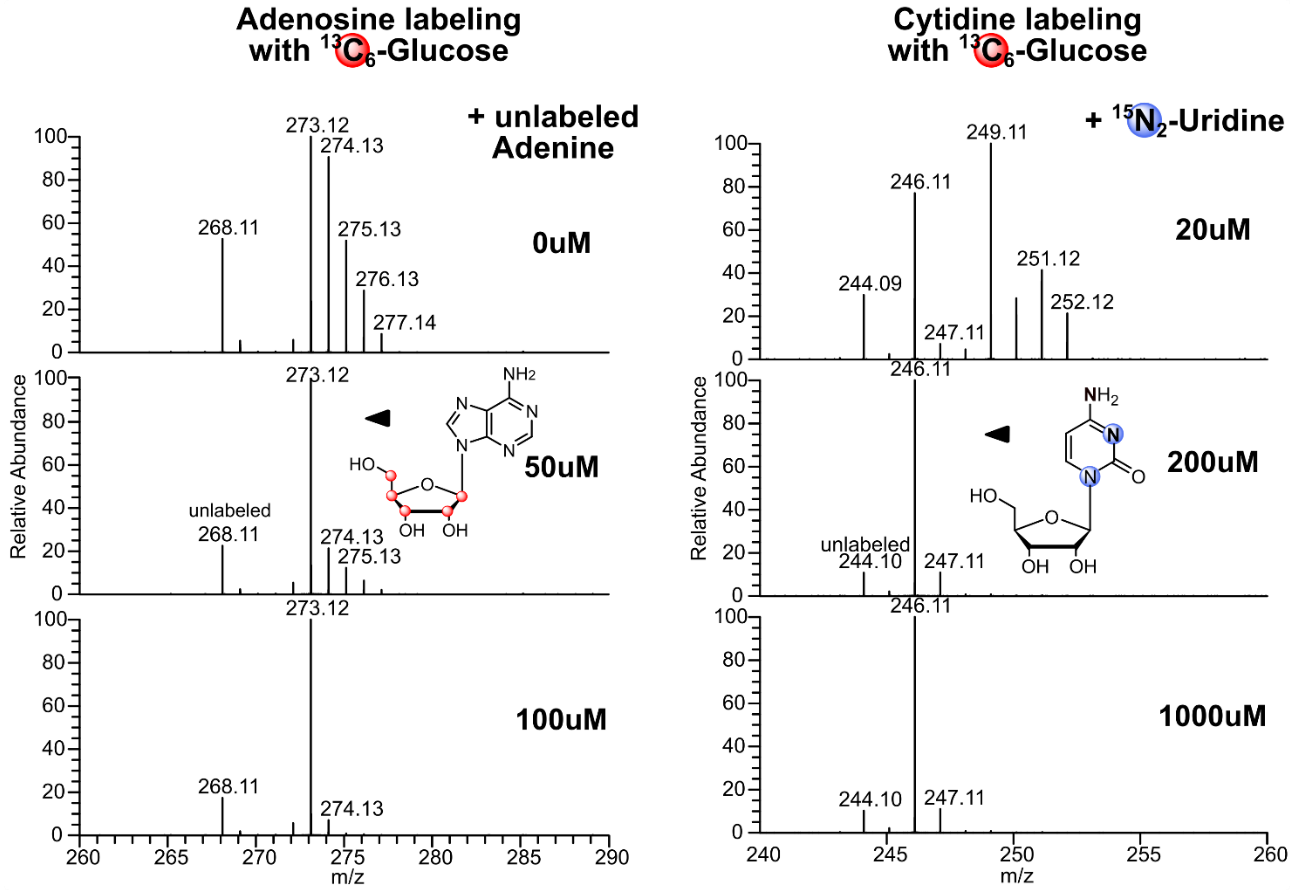

Figure S6: Concentration optimization of adenine and uridine for stable isotope labeling. The high-resolution mass spectra on the left show stable isotope labeling of adenosine after labeling with $^{13}C_6$-glucose and different concentrations of unlabeled adenine. The high-resolution spectra on the right show stable isotope labeling of cytidine after labeling with $^{13}C_6$-glucose and different concentrations of $^{15}N_2$-uridine. All cells were labeled for 7 days (=3 passages) in the respective medium. Note: Unlabeled signals are caused by the low labeling efficiency in $^{13}C_6$-glucose medium.

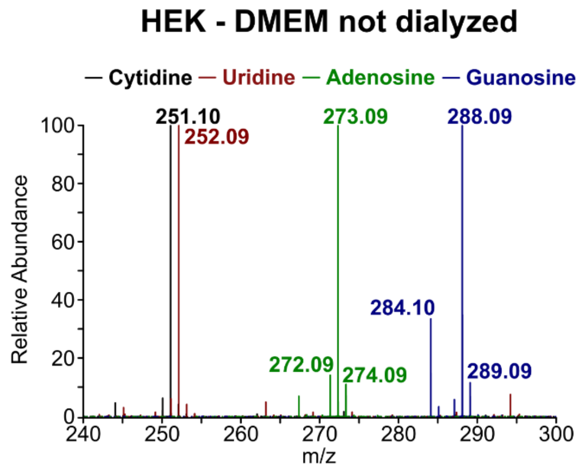

Figure S7: Stable isotope labeling of nucleosides with labeled uridine and adenine using undialyzed FBS.

Merged high-resolution mass spectra of cytidine, uridine, guanosine and adenosine after stable isotope labeling of HEK 293 cell culture using DMEM D0422 supplemented with $^{13}C_5$,$^{15}N_2$-uridine and $^{15}N_5$-adenine but normal FBS instead of dialyzed FBS. Cells were labeled for 7 days (=3 passages).

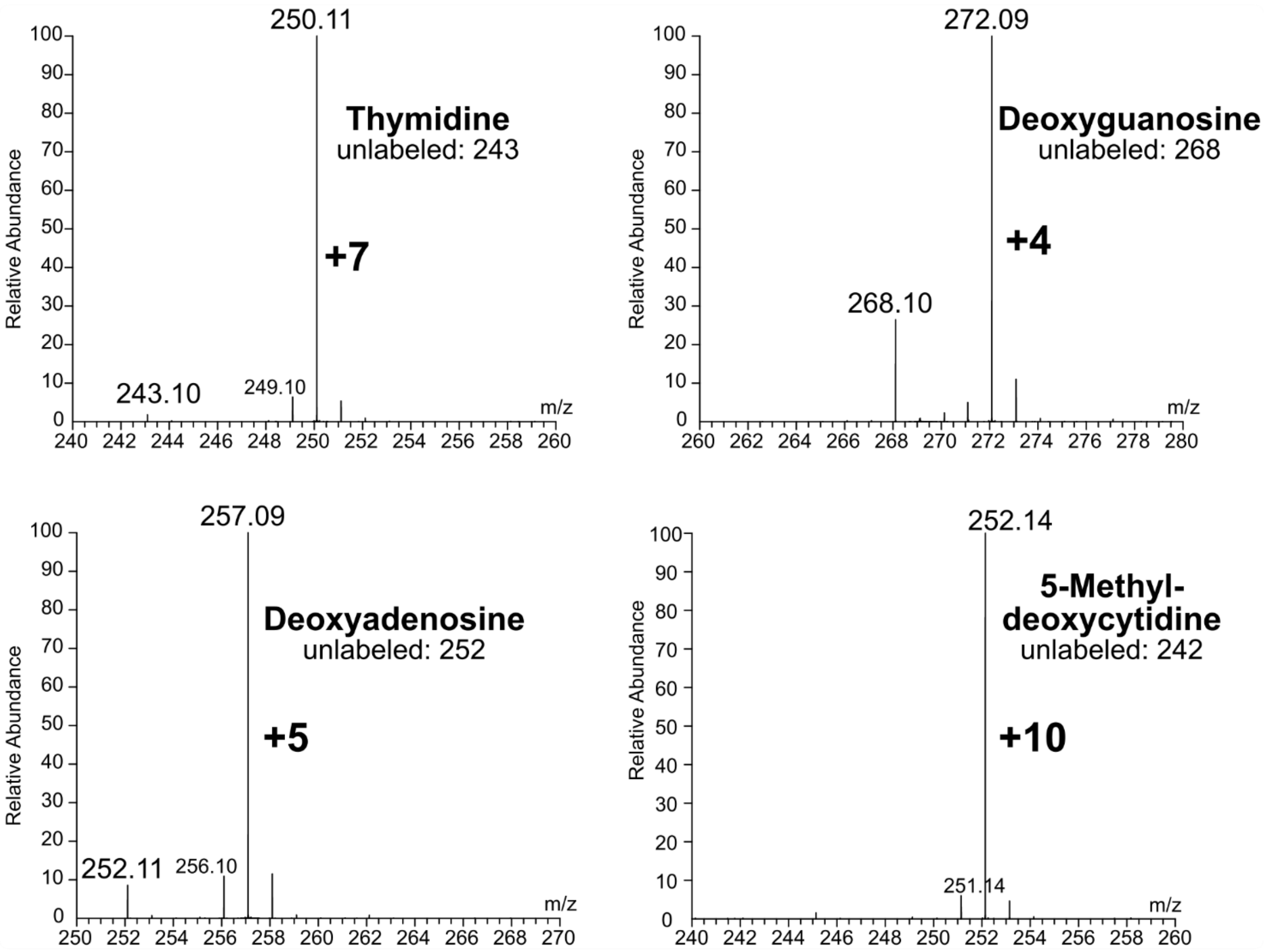

Figure S8: Stable isotope labeling of DNA

High-resolution mass spectra of DNA nucleosides after labeling in DMEM D0422 medium supplemented with stable isotope labeled $^{15}N_5$-adenine, $^{13}C_5{}^{15}N_2$-uridine and CD_3-methionine. Cells were labeled for 7 days (=3 passages) and DNA was purified (mini spin columns) and digested to nucleosides using standard procedures.

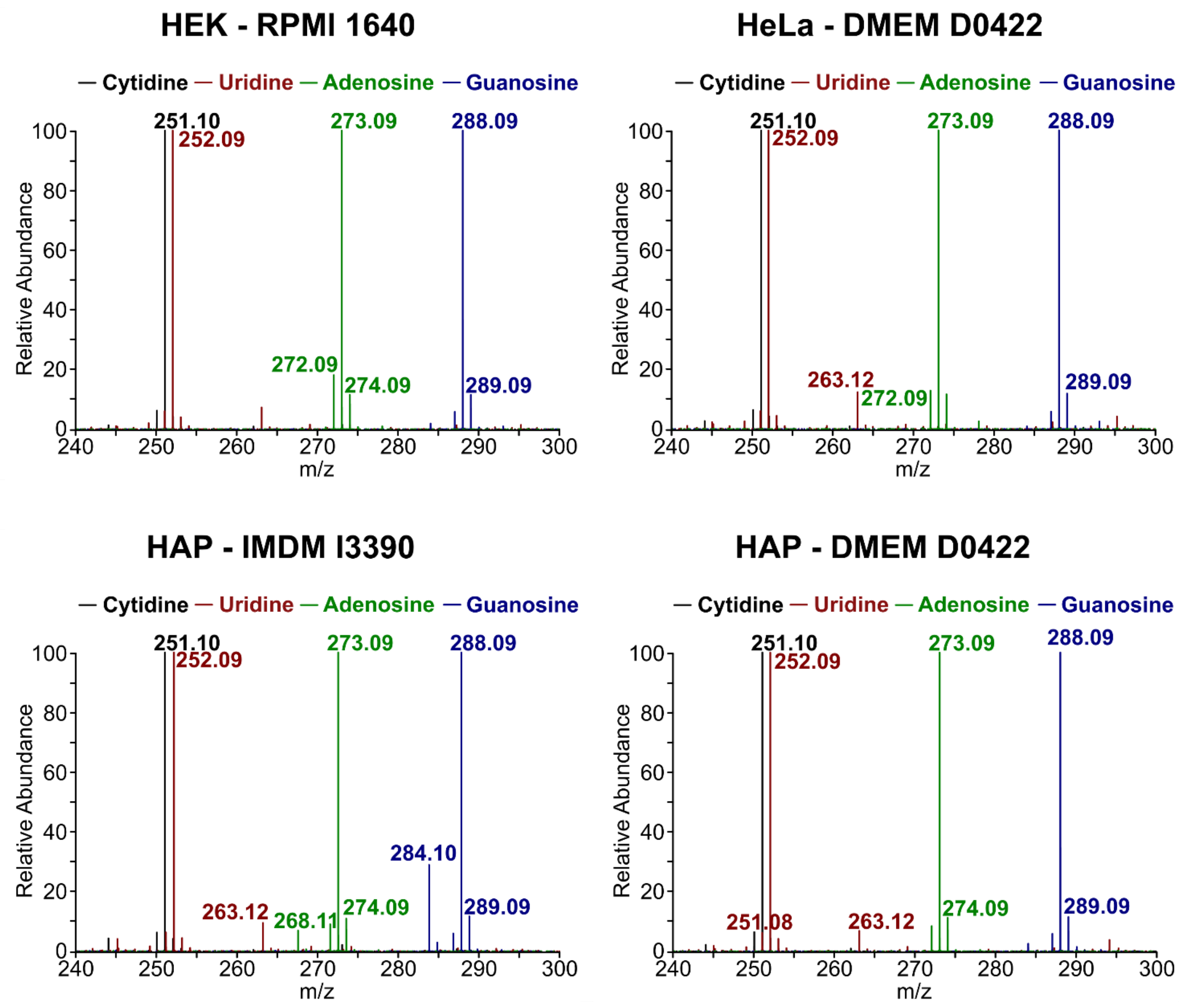

Figure S9: Stable isotope labeling of different cell lines in different media.
Merged high-resolution spectra of cytidine, uridine, guanosine and adenosine after stable isotope labeling of different cell lines using the respective medium supplemented with stable isotope labeled $^{15}N_5$-adenine and $^{13}C_5$,$^{15}N_2$-uridine and dialyzed FBS. All cells were labeled for 7 days (=3 passages) in the respective medium.

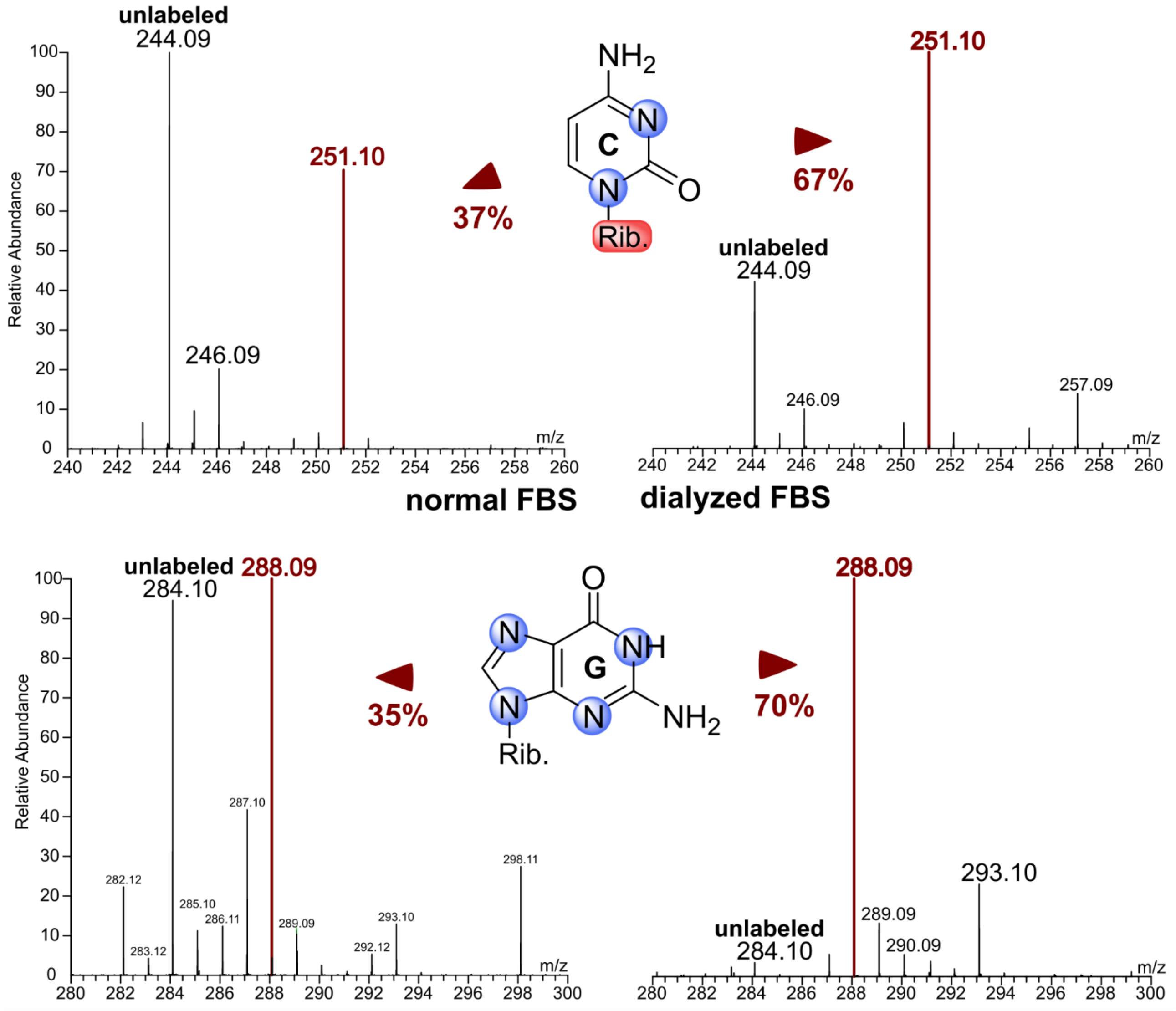

Figure S10: Labeling of mESC RNA.

The high-resolution mass spectra on the left show stable isotope labeling of cytidine (top) and guanosine (bottom) after labeling mouse embryonic stem cells with $^{15}N_5$-adenine and $^{13}C_5{}^{15}N_2$-uridine in DMEM containing FBS and LIF (leukemia inhibitory factor) for 4 days (for detailed information see Rahimoff *et al.* [1]. The percentages were calculated by adding up the relative abundances of all peaks for the nucleoside of interest. The relative abundance of the peak with desired labeling was then divided by the sum of all peaks.

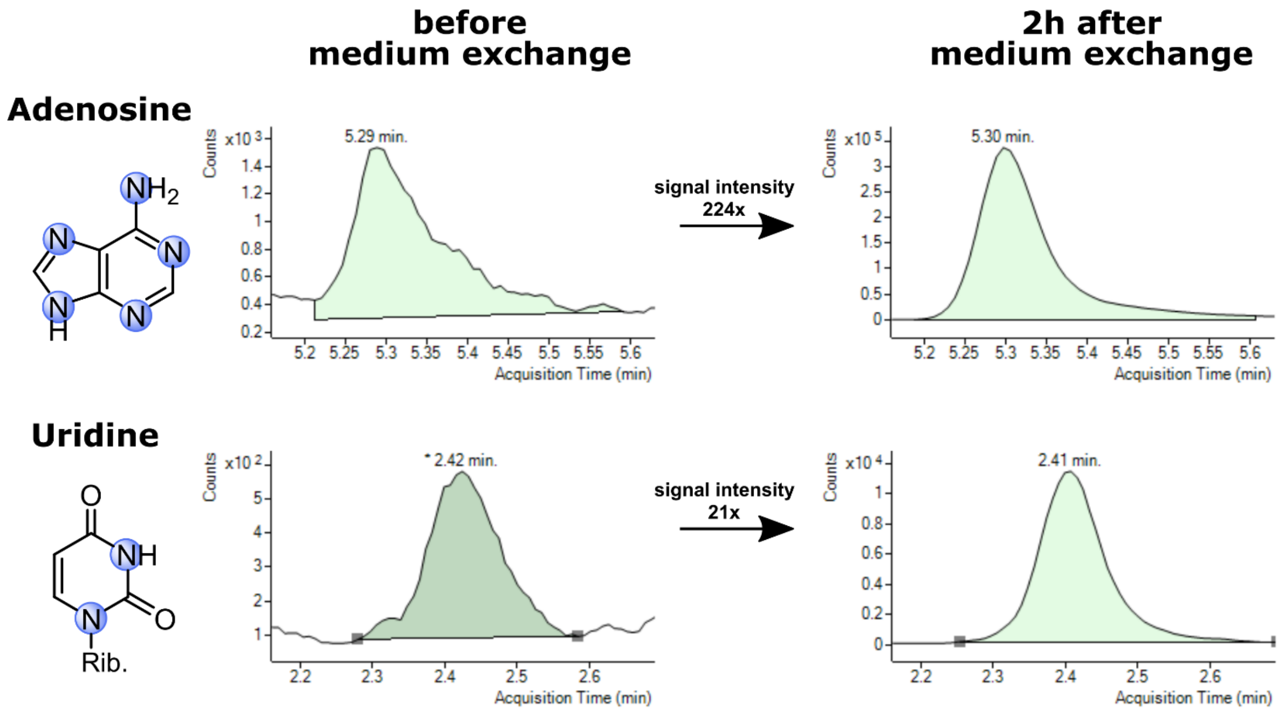

Figure S11: Occurrence of labeled nucleosides 2 hours after switch to labeled growth media. MS signals of labeled adenosine (top, 273 → 136) and uridine (bottom, 247 →115) without stable isotope labeling are shown on the left. MS signals of labeled adenosine and uridine after labeling for 2 hours are shown on the right.

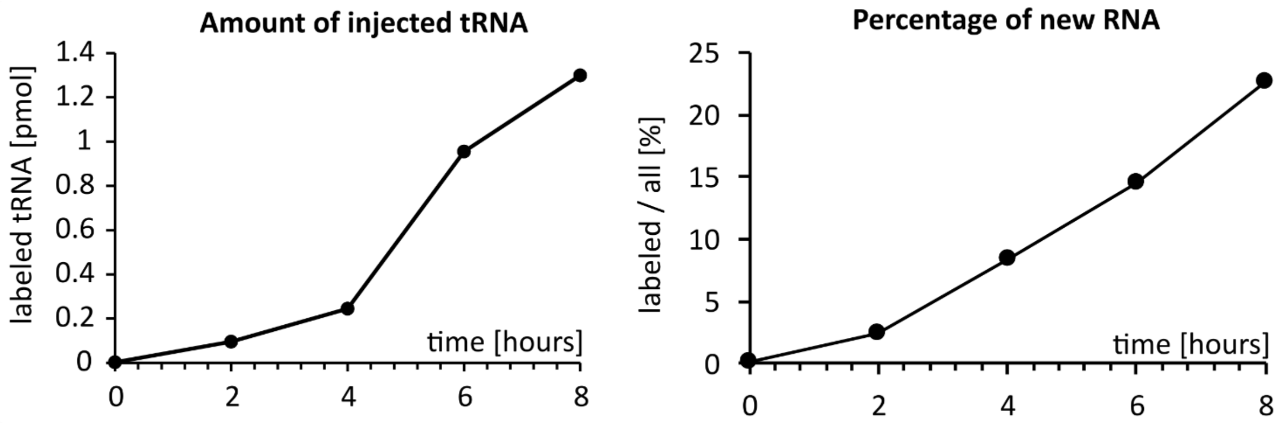

Figure S12: Increase of labeled tRNA molecules.
For all time points the same mass of RNA was injected (according to UV measurements of undigested RNA using an IMPLEN Nanophotometer, Munich, Germany). The injected amount of tRNA was calculated based on the sum of measured values for C, U, G and A. Left: Absolute amount of labeled tRNA. Right: Relative increase of labeled tRNA in proportion to all tRNA (unlabeled + labeled).

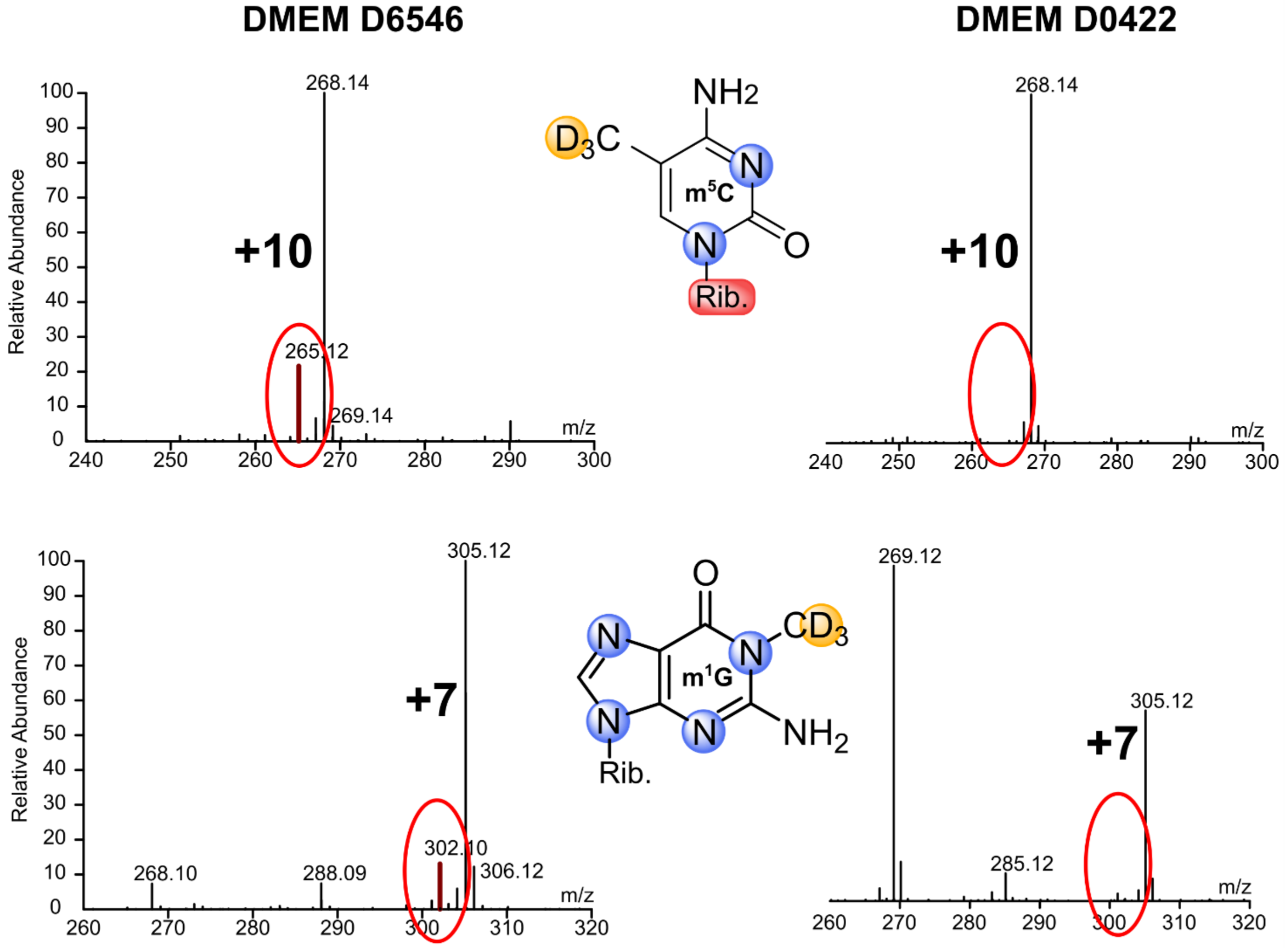

Figure S13: Labeling of methyl-groups by D_3-methionine.
Left: High-resolution mass spectra of m^5C and m^1G after labeling in DMEM D6546 supplemented with stable isotope labeled $^{15}N_5$-adenine and $^{13}C_5{}^{15}N_2$-uridine and 0.15 g/L (5x) D_3-methionine. Right: High-resolution mass spectra of m^5C and m^1G after labeling in DMEM D0422 supplemented with stable isotope labeled $^{15}N_5$-adenine and $^{13}C_5{}^{15}N_2$-uridine and 0.03 g/L (1x) D_3-methionine. All cells were labeled for 7 days (=3 passages) in the respective medium. Red circles highlight the signal for $^{13}C/^{15}N$-labeled nucleosides with an undesired CH_3-methylation.

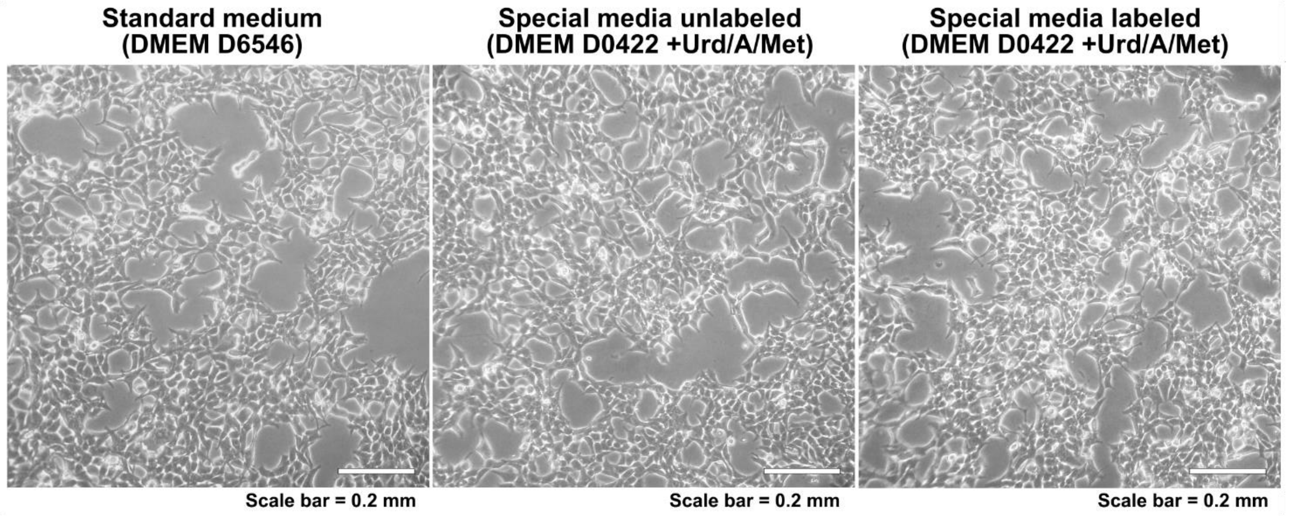

Figure S14: Photographs of HEK 293 cells grown in different DMEM media.
1.2 million cells were plated in a T25 flask and cultured in the respective media for 2 days. DMEM D6546 was supplemented with glutamine and FBS only. DMEM D0422 was supplemented with glutamine, FBS and cystine. Methionine, adenine and uridine were supplemented in DMEM D0422 as unlabeled or labeled compounds respectively. Before experiments, cell shape and size were inspected regularly and always corresponded to this phenotype.

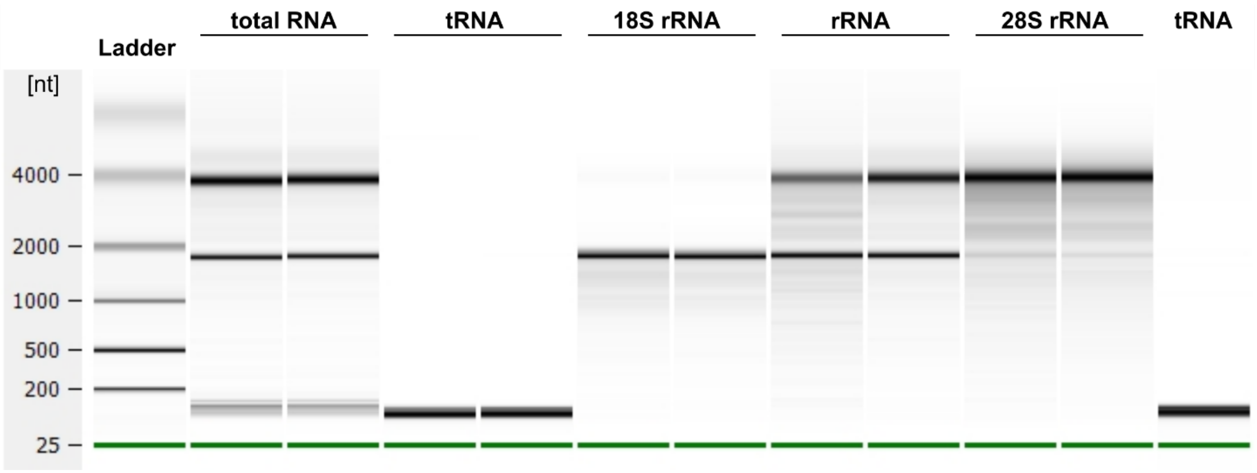

Figure S15: BioAnalyzer Pico chip of SEC purified RNA species.
For each species the exemplary timepoint = 0 of a forward and a reverse experiment are shown. Total RNA, 18S rRNA, total rRNA and 28S rRNA are from samples shown in Fig. 3. tRNA is from samples shown in Fig. 4 and was used for purification of $tRNA^{Phe}$. tRNA in the right lane was used for purification of $tRNA^{Phe}$ for Fig. 1. The reproducibility of RNA purification was confirmed as high by several similar experiments.

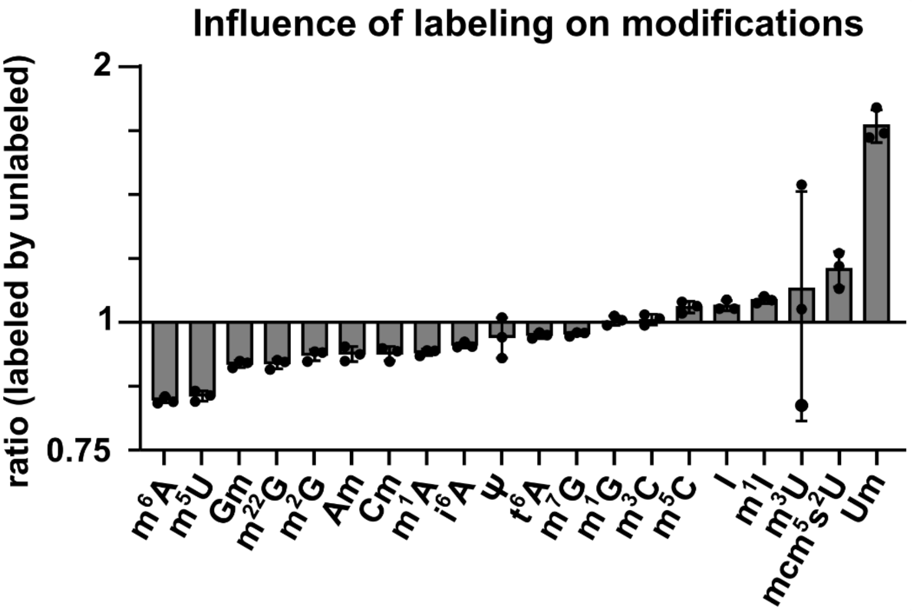

Figure S16: Ratio of labeled to unlabeled modification amount in the mix samples.
Cells were cultured for 7 days (=3 passages) in unlabeled or labeled media. After harvesting, the cell suspensions were mixed for subsequent co-processing. Total tRNA was purified and digested to nucleosides using standard procedures. For each modification the calculated amount of labeled modification per labeled tRNA molecule was divided by the amount of unlabeled modification per unlabeled tRNA molecule respectively. The experiment was done in n = 3 biol. replicates. Bars reflect the mean and error bars reflect standard deviation.

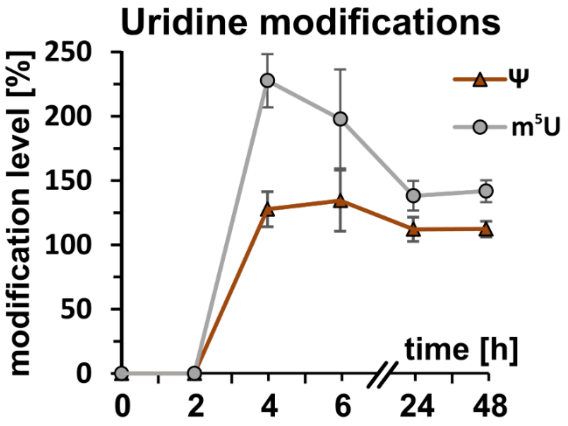

Figure S17: Occurrence of pseudouridine (Ψ) and 5-methyluridine (m^{5}U) in new transcripts.
Cells were grown in unlabeled DMEM D0422 (+uridine, + adenine) for 7 days. At T = 0 the medium was exchanged to DMEM D0422 supplemented with $^{15}N_5$-adenine and $^{13}C_5{}^{15}N_2$-uridine. Cells were harvested after set time points and tRNA^{Phe} was purified and analyzed by LC-MS/MS. Plotted on the y-axis is the level of modification in new transcripts where 100% equals the amount of the respective nucleoside originating from unlabeled medium before experiment initiation (T = 0). The experiment was done in n = 3 biol. replicates. Symbols reflect the mean and error bars reflect standard deviation.

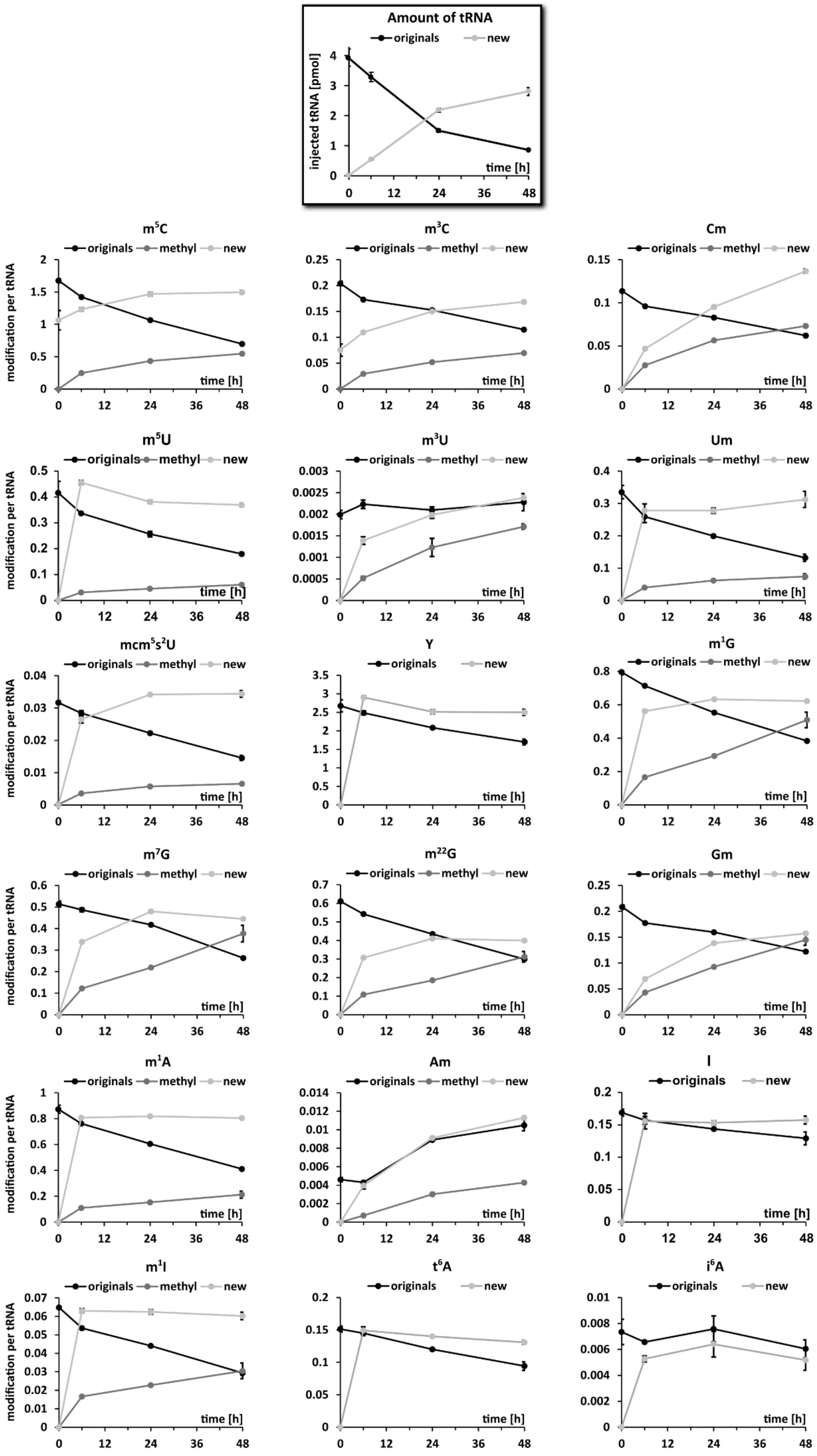

Figure S18: Maturation processes of total tRNA in detail.

Original nucleosides (originals, black line) already existed before experiment initiation. Post-methylated nucleosides (methyl, dark grey line) are modifications arising from the methylation of original RNA after experiment initiation. New nucleosides (new, light grey line) show the incorporation of modification into new transcripts. Data points reflect the mean and standard deviations of n = 3 biol. replicates.

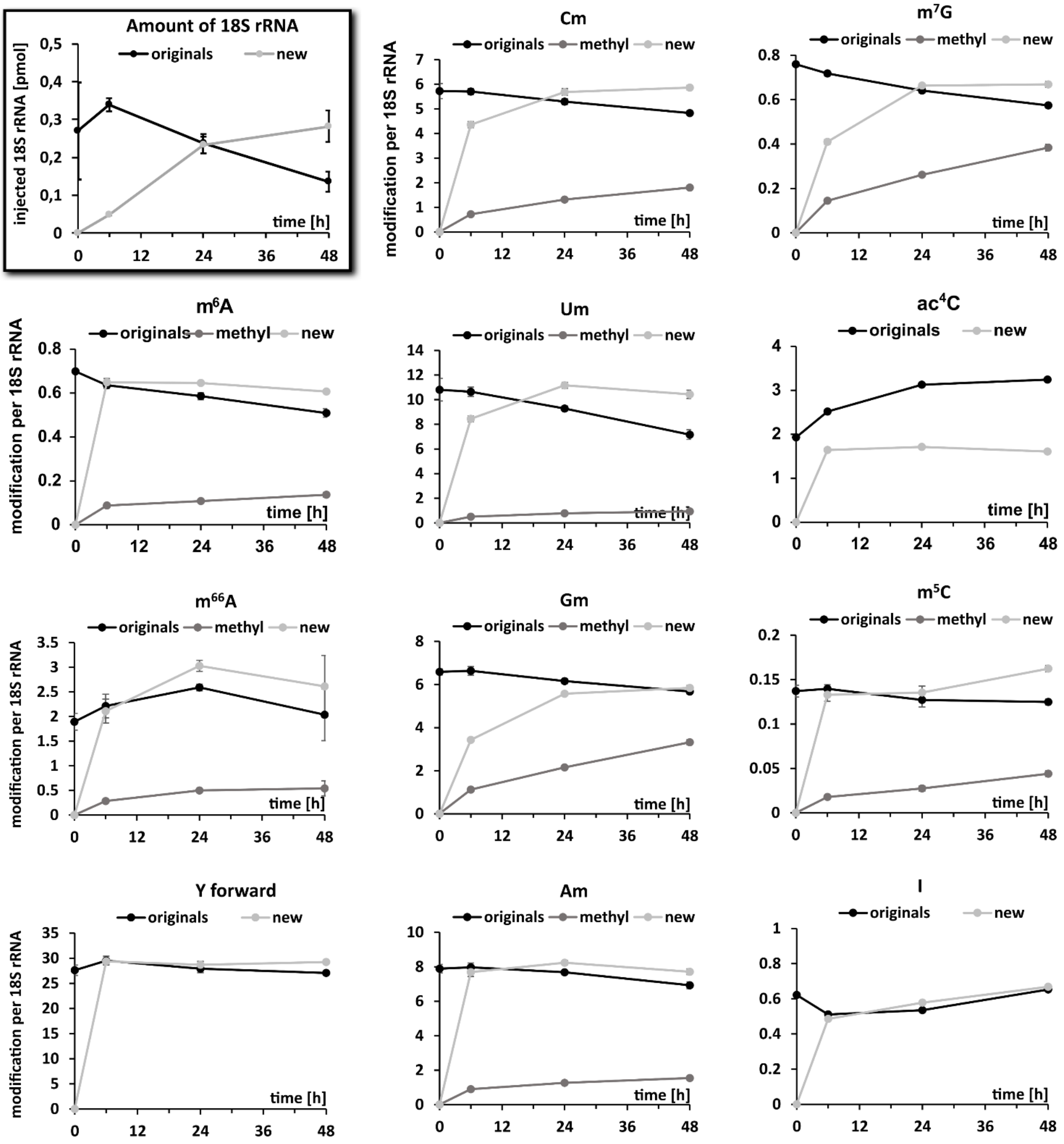

Figure S19: Maturation processes of 18S rRNA in detail.
Original nucleosides (originals, black line) already existed before experiment initiation. Post-methylated nucleosides (methyl, dark grey line) are modifications arising from the methylation of original RNA after experiment initiation. New nucleosides (new, light grey line) show the incorporation of modification into new transcripts. Data points reflect the mean and standard deviations of n = 3 biol. replicates.

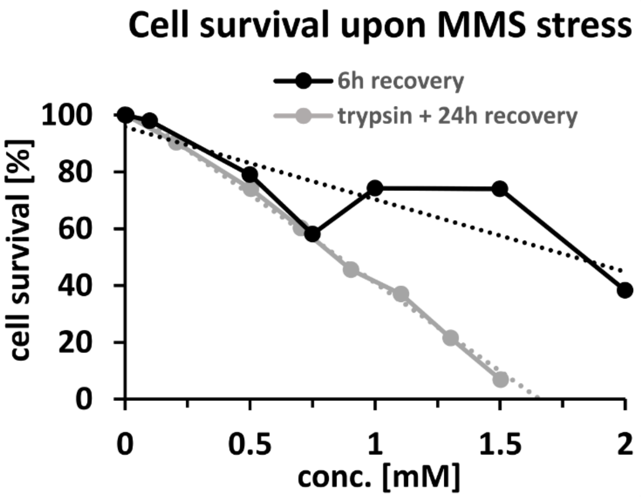

Figure S20: Effect of MMS on growth of HEK 293 cells.
HEK 293 cells were grown to ~70% confluency in DMEM D0422 medium supplemented with unlabeled uridine and adenine. The respective concentration of MMS was then added by exchanging the medium. After 1h the MMS containing medium was removed again and substituted by the starting medium for recovery. After 6h alive cells were counted using trypan blue and a hemocytometer (black line). For 24 h recovery (grey line) the stress medium was first removed followed by trypsinization and seeding of the cells on a new plate (1:2 split). After 24 h living cells were counted using trypan blue and a hemocytometer. The dashed lines represent the respective regression curves.

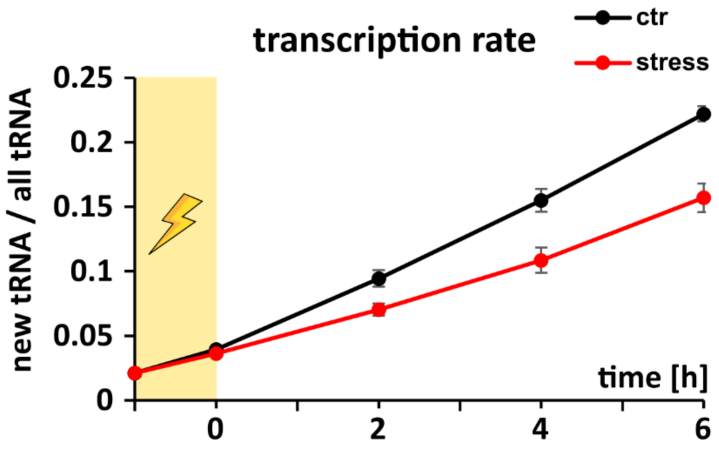

Figure S21: Transcription rate of stressed cells.
70% confluent D_3-labeled cells were incubated with fully labeled media (stable isotope labeled $^{15}N_5$-adenine, $^{13}C_5{}^{15}N_2$-uridine and CD_3-methionine) for 2 h before the LD_{50} dose of methyl methanesulfonate (MMS, yellow shaded area) was added (T = −1). After 1 h the stress media was replaced by fresh fully labeled media. After set time points (0 h, 2 h, 4 h, 6 h) the cells were harvested and $tRNA^{Phe}_{GAA}$ was purified and subjected to LC-MS/QQQ analysis. Isoacceptor purification and digestion to nucleosides was done using standard procedures. The sum of new canonical nucleosides (labeled) was divided by the sum of all canonical nucleosides (unlabeled + labeled) and plotted against the time points for the control and stressed cells respectively. All experiments are from n = 3 biol. replicates. Symbols reflect the mean and error bars reflect standard deviation.

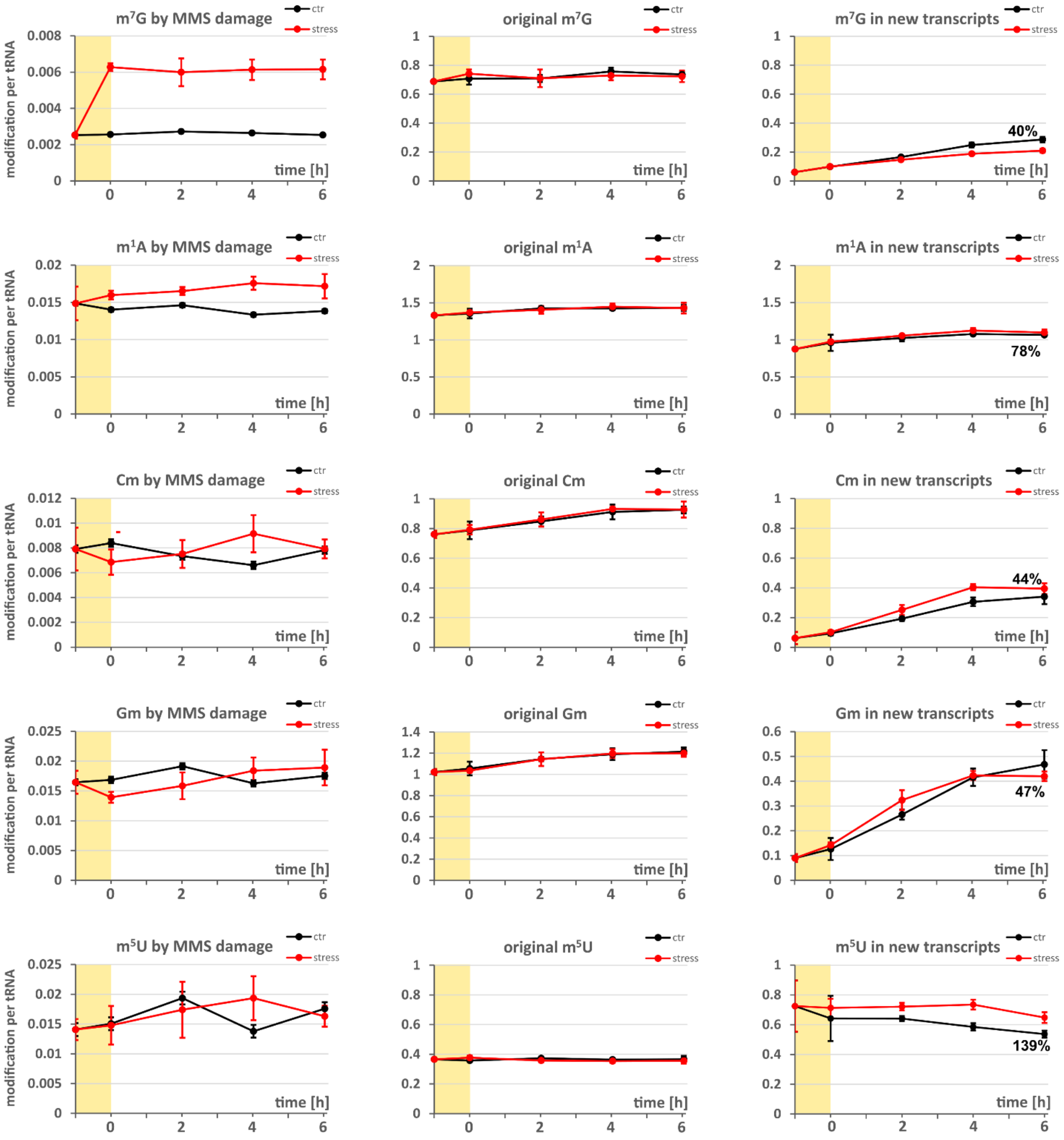
m^{7}G by MMS damage
original m^{7}G
m^{7}G in new transcripts
40%
m^{1}A by MMS damage
original m^{1}A
m^{1}A in new transcripts
78%
Cm by MMS damage
original Cm
Cm in new transcripts
44%
Gm by MMS damage
original Gm
Gm in new transcripts
47%
m^{5}U by MMS damage
original m^{5}U
m^{5}U in new transcripts
139%
ctr
stress
modification per tRNA
time [h]

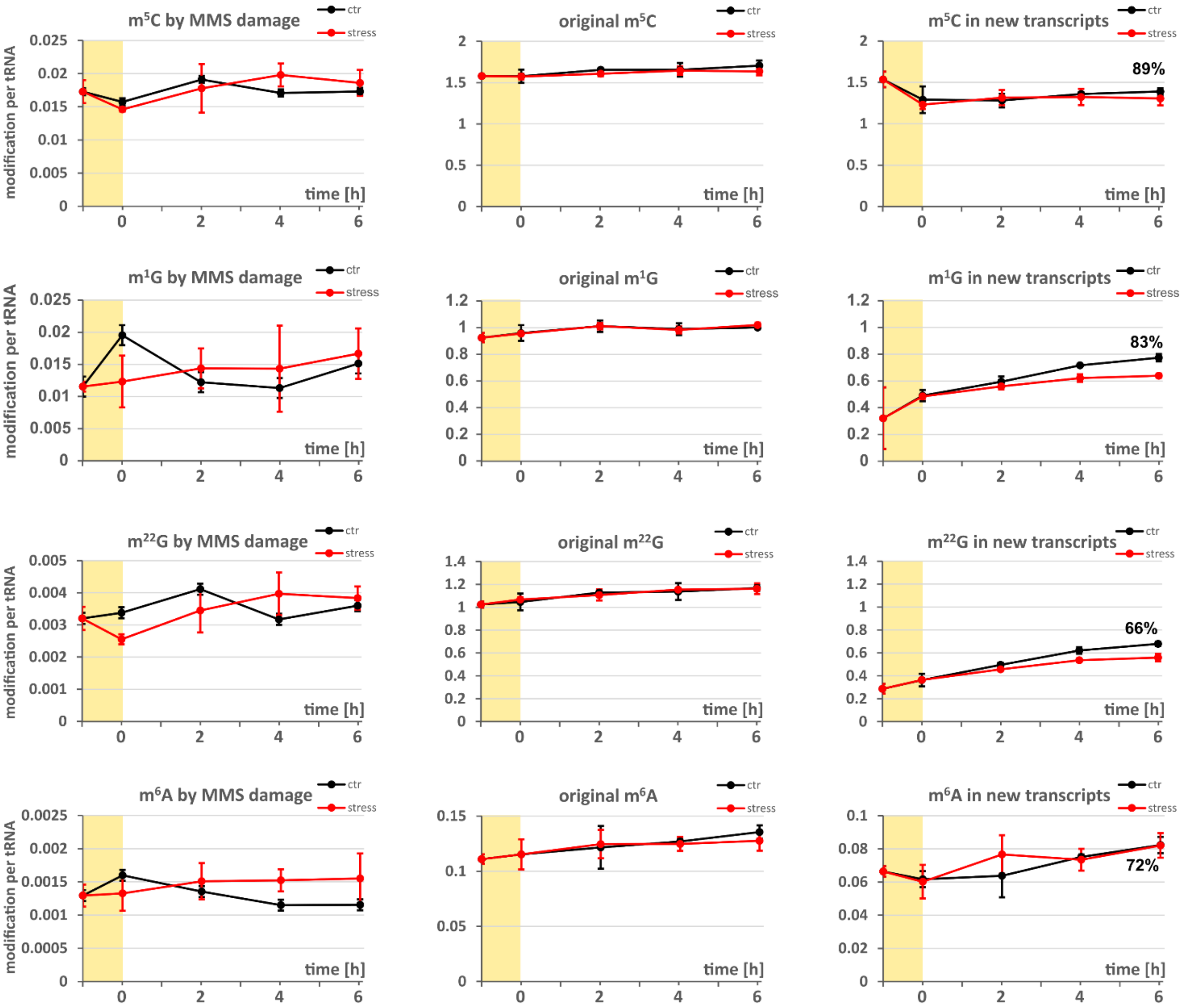

Figure S22: Effect of MMS on tRNA modification dynamics

70% confluent D_3-labeled cells were incubated with fully labeled media for 2 h before the LD_{50} dose of methyl methanesulfonate (MMS, yellow shaded area) was added (T = −1). After 1 h the stress media was replaced by fresh labeled media. After set time points (0 h, 2 h, 4 h, 6 h) the cells were harvested and $tRNA^{Phe}_{GAA}$ was purified and subjected to LC-MS/QQQ analysis. Left: Modification per tRNA molecule arising from direct MMS damage in control and MMS stressed cells. Unlabeled modifications were referenced to unlabeled canonicals to calculate the amount of modifications arising from direct methylation damage by MMS. Middle: Modification per tRNA molecule in original transcripts (already existent before medium exchange at T = −1) in control and MMS stressed cells. D_3-labeled modifications were referenced to unlabeled canonicals to calculate the amount of modifications in original transcripts. Right: Modification per tRNA molecule in new transcripts. Labeled modifications were referenced to labeled canonicals to calculate the amount of modifications in new tRNA transcripts. The numbers at time point 6 give the percentage of modification amount in the control sample referenced to the naturally occurring amount of the respective modification (T = −1). The experiment was done in n = 3 biol. replicates. Bars reflect the mean and error bars reflect standard deviation.

Table S1: Results of scanned modifications for quantification of $tRNA^{Phe}$.
The values are calculated from n = 3 biological triplicates and give the number of modifications per average $tRNA^{Phe}$ molecule. The average is calculated by the mean. n.d., not detectable. 2-Methylguanosine (m^2G), although detectable in reasonable amount was excluded from this experiment due to problems with accurate MS quantification for this modification.

Modification	Average	Standard deviation
Y	4.436	0.027
D	3.754	0.172
m^1A	1.464	0.015
m^5C	1.236	0.014
$m^{22}G$	1.042	0.005
Gm	0.990	0.008
Cm	0.909	0.006
m^7G	0.720	0.011
m^5U	0.479	0.011
m^6A	0.313	0.003
m^1G	0.278	0.009
I	0.063	0.003
m^1I	0.026	0.004
t^6A	0.016	0.004
i^6A	0.006	0.004
m^3C	n.d.	
m^3U	n.d.	
Am	n.d.	
Um	n.d.	
mcm^5s^2U	n.d.	
mcm^5U	n.d.	
ncm^5U	n.d.	
m^1Y	n.d.	

Table S2: Sequences of DNA oligonucleotide probes used for Northern Blot analysis.
Cyanine-3 (Cy3) was attached to both ends of the oligonucleotides to enhance signal intensity. IVT = *in vitro*, tRF = tRNA fragment. All oligonucleotides were designed using the modomics database and ordered from Sigma-Aldrich, Munich, Germany.

Probe against:	Sequence of oligonucleotide
$tRNA^{Phe}_{GAA}$	(Cy3)-TGGTGCCGAAACCCGGGATCGAACCAGGGT-(Cy3)
IVT $tRNA^{Ser}_{UGA}$	(Cy3)-TTTCAAGTCCATCGCCTTAACCACTCGGCCACGACTAC-(Cy3)
tRF Gly_{GCC}	(Cy3)-CAGGCGAGAATTCTACCACTGAACCACCAATGC-(Cy3)
5S rRNA	(Cy3)-AAACCGACCCTGCTTAGCTTCCGAGATCAGACG-(Cy3)

Table S3: Parameters for each measured compound of MRM methods for QQQ analysis. Nucleosides are abbreviated with the common code found at modomics [2]. “Unlabeled” refers to nucleosides from unlabeled medium; “nucleoside body labeled” refers to nucleosides from $^{15}N_5$-adenine and $^{13}C_5{}^{15}N_2$-uridine medium; “methyl labeled” refers to nucleosides with no label of nucleobase or sugar but CD_3-methylation; “nucleoside body and methyl labeled” refers to nucleosides grown in the presence of $^{15}N_5$-adenine, $^{13}C_5{}^{15}N_2$-uridine and CD_3-methionine; “SILIS” is short for stable isotope labeled internal standard and was produced in yeast using a rich $^{15}N/^{13}C$ growth medium following our published procedure [3].

	Compound Name	Precursor Ion	Product Ion	Ret Time (min)	Fragmentor (V)	Collision Energy (eV)
unlabeled	A	268	136	5,2	200	20
	acp^{3}U	346	214	2,3	95	15
	Am	282	136	6,0	130	17
	C	244	112	2,1	200	20
	Cm	258	112	4,1	180	9
	D	247	115	1,6	70	5
	G	284	152	4,3	200	20
	Gm	298	152	5,0	100	9
	I	269	137	4,1	100	10
	i^{6}A	336	204	8,0	140	17
	m^{1}A	282	150	2,5	150	25
	m^{1}G	298	166	4,9	105	13
	m^{1}I	283	151	4,8	80	12
	m^{1}Y	259	223	3,1	85	5
	m^{22}G	312	180	5,7	105	13
	m^{2}G	298	166	5,1	95	17
	m^{3}C	258	126	2,3	88	14
	m^{3}U	259	127	4,8	75	9
	m^{5}C	258	126	3,8	185	13
	m^{5}U	259	127	4,4	95	9
	m^{6}A	282	150	6,5	125	17
	m^{7}G	298	166	3,6	100	13
	mcm^{5}s^{2}U	333	201	6,2	92	8
	t^{6}A	413	281	5,8	130	9
	U	245	113	3,0	95	5
	Um	259	113	4,6	96	8
	Y	245	209	1,7	90	5
	mcm^{5}U	317	185	5,0	95	5
	ncm^{5}U	302	170	2,5	85	8

	Compound Name	Precursor Ion	Product Ion	Ret Time (min)	Fragmentor (V)	Collision Energy (eV)
nucleoside body labeled	A lab	273	141	5,2	200	20
	acp^{3}U lab	353	216	2,3	95	15
	Am lab	287	141	6,0	130	17
	C lab	251	114	2,1	200	20
	Cm lab	265	114	4,1	180	9
	D lab	254	117	1,6	70	5
	G lab	288	156	4,3	200	20
	Gm lab	302	156	5,0	100	9
	I lab	273	141	4,1	100	10
	i^{6}A lab	341	209	8,0	140	17
	m^{1}A lab	287	155	2,5	150	25
	m^{1}G lab	302	170	4,9	105	13
	m^{1}I lab	287	155	4,8	80	12
	m^{1}Y lab	266	230	3,1	85	5
	m^{22}G lab	316	184	5,7	105	13
	m^{2}G lab	302	170	5,1	95	17
	m^{3}C lab	265	128	2,3	88	14
	m^{3}U lab	266	129	4,8	75	9
	m^{5}C lab	265	128	3,8	185	13
	m^{5}U lab	266	129	4,4	95	9
	m^{6}A lab	287	155	6,5	125	17
	m^{7}G lab	302	170	3,6	100	13
	mcm^{5}s^{2}U lab	340	203	6,2	92	8
	t^{6}A lab	418	286	5,8	130	9
	U lab	252	115	3,0	95	5
	Um lab	266	115	4,6	96	8
	Y lab	252	216	1,7	90	5
	mcm^{5}U lab	324	187	5,0	95	5
	ncm^{5}U lab	309	172	2,5	85	8
methyl labeled	Am D_3	285	136	6,0	130	17
	Cm D_3	261	112	4,1	180	9
	Gm D_3	301	152	5,0	100	9
	m^{1}A D_3	285	153	2,5	150	25
	m^{1}G D_3	301	169	4,9	105	13
	m^{1}I D_3	286	154	4,8	80	12
	m^{1}Y D_3	262	226	3,1	85	5
	m^{22}G D_3	318	186	5,7	105	13
	m^{2}G D_3	301	169	5,1	95	17
	m^{3}C D_3	261	129	2,3	88	14
	m^{3}U D_3	262	130	4,8	75	9

	Compound Name	Precursor Ion	Product Ion	Ret Time (min)	Fragmentor (V)	Collision Energy (eV)
	m^{5}C D$_3$	261	129	3,8	185	13
	m^{5}U D$_3$	262	130	4,4	95	9
	m^{6}A D$_3$	285	153	6,5	125	17
	m^{7}G D$_3$	301	169	3,6	100	13
	mcm^{5}s^{2}U D$_3$	336	204	6,2	92	8
	Um D$_3$	262	113	4,6	96	8
	mcm^{5}U D$_3$	320	188	5,0	95	5
nucleoside body and methyl labeled	Am lab D$_3$	290	141	6,0	130	17
	Cm lab D$_3$	268	114	4,1	180	9
	Gm lab D$_3$	305	156	5,0	100	9
	m^{1}A lab D$_3$	290	158	2,5	150	25
	m^{1}G lab D$_3$	305	173	4,9	105	13
	m^{1}I lab D$_3$	290	158	4,8	80	12
	m^{1}Y lab D$_3$	269	233	3,1	85	5
	m^{22}G lab D$_3$	322	190	5,7	105	13
	m^{2}G lab D$_3$	305	173	5,1	95	17
	m^{3}C lab D$_3$	268	131	2,3	88	14
	m^{3}U lab D$_3$	269	132	4,8	75	9
	m^{5}C lab D$_3$	268	131	3,8	185	13
	m^{5}U lab D$_3$	269	132	4,4	95	9
	m^{6}A lab D$_3$	290	158	6,5	125	17
	m^{7}G lab D$_3$	305	173	3,6	100	13
	mcm^{5}s^{2}U lab D$_3$	343	206	6,2	92	8
	Um lab D$_3$	269	115	4,6	96	8
	mcm^{5}U lab D$_3$	327	190	5,0	95	5
SILIS	A SILIS	283	146	5,2	200	20
	Am SILIS	298	146	6,0	130	17
	C SILIS	256	119	2,1	200	20
	Cm SILIS	271	119	4,1	180	9
	D SILIS	258	121	1,6	70	5
	G SILIS	299	162	4,3	200	20
	Gm SILIS	314	162	5,0	100	9
	I SILIS	283	146	4,1	100	10
	i^{6}A SILIS	356	219	8,0	140	17
	m^{1}A SILIS	298	161	2,5	150	25
	m^{1}G SILIS	314	177	4,9	105	13
	m^{1}I SILIS	298	161	4,8	80	12
	m^{22}G SILIS	329	192	5,7	105	13

	Compound Name	Precursor Ion	Product Ion	Ret Time (min)	Fragmentor (V)	Collision Energy (eV)
	m^2G SILIS	314	177	5,1	95	17
	m^3C SILIS	271	134	2,3	88	14
	m^5C SILIS	271	134	3,8	185	13
	m^5U SILIS	271	134	4,4	95	9
	m^6A SILIS	298	161	6,5	125	17
	m^7G SILIS	314	177	3,6	100	13
	mcm^5s^2U SILIS	347	210	6,2	92	8
	t^6A SILIS	434	297	5,8	130	9
	U SILIS	256	119	3,0	95	5
	Um SILIS	271	119	4,6	96	8
	Y SILIS	256	220	1,7	90	5
	mcm^5U SILIS	331	194	5,0	95	5
	ncm^5U SILIS	316	179	2,5	85	8

Table S4: Quantification of m^7G per tRNA (based on G)

First the molar amount of injected nucleosides is calculated based on the signal areas of target nucleosides and SILIS and the respective rRFNs determined by calibration (here for m7G and G). Then the molar amount of modification is divided by the molar amount of respective tRNA calculated by dividing the molar amount of canonical by the expected number of the respective canonical (here just based on G).

	m^7G [pmol]	G [pmol]	m^7G per tRNA
original	$\frac{\text{area } m^7G \text{ (unlabeled)}}{\text{rRFN } m^7G \bullet \text{area } m^7G \text{ (SILIS)}}$	$\frac{\text{area G (unlabeled)}}{\text{rRFN G} \bullet \text{area G (SILIS)}}$	$\frac{m^7G \text{ (original)}}{\frac{\text{G (original)}}{\# \, of \, G \, in \, sequence}}$
new	$\frac{\text{area } m^7G \, (^{15}N, CD_3)}{\text{rRFN } m^7G \bullet \text{area } m^7G \text{ (SILIS)}}$	$\frac{\text{area G } (^{15}N)}{\text{rRFN G} \bullet \text{area G (SILIS)}}$	$\frac{m^7G \text{ (new)}}{\frac{\text{G (new)}}{\# \, of \, G \, in \, sequence}}$

References:

1. Rahimoff, R. et al. 5-Formyl- and 5-Carboxydeoxycytidines Do Not Cause Accumulation of Harmful Repair Intermediates in Stem Cells. *J Am Chem Soc* **139**, 10359-10364 (2017).
2. Boccaletto, P. et al. MODOMICS: a database of RNA modification pathways. 2017 update. *Nucleic Acids Res* **46**, D303-D307 (2018).
3. Borland, K. et al. Production and Application of Stable Isotope-Labeled Internal Standards for RNA Modification Analysis. *Genes (Basel)* **10** (2019).
