# Supplementary material for: Cell culture NAIL-MS allows insight into human tRNA and rRNA modification dynamics in vivo
Source: Nat Commun. 2021 Jan 15;12:389. doi: 10.1038/s41467-020-20576-4 (PMC7810713; doi:10.1038/s41467-020-20576-4)
Supplement: Supplementary file 2 — Reporting Summary [file 41467_2020_20576_MOESM2_ESM.pdf]

nature research

Corresponding author(s): Stefanie Kellner

Last updated by author(s): Nov 11, 2020

Reporting Summary

Nature Research wishes to improve the reproducibility of the work that we publish. This form provides structure for consistency and transparency in reporting. For further information on Nature Research policies, see Authors & Referees and the Editorial Policy Checklist .

Statistics

For all statistical analyses, confirm that the following items are present in the figure legend, table legend, main text, or Methods section.

n/a	Confirmed	
☐	☒	The exact sample size (n) for each experimental group/condition, given as a discrete number and unit of measurement
☐	☒	A statement on whether measurements were taken from distinct samples or whether the same sample was measured repeatedly
☐	☒	The statistical test(s) used AND whether they are one- or two-sided<br>*Only common tests should be described solely by name; describe more complex techniques in the Methods section.*
☒	☐	A description of all covariates tested
☒	☐	A description of any assumptions or corrections, such as tests of normality and adjustment for multiple comparisons
☐	☒	A full description of the statistical parameters including central tendency (e.g. means) or other basic estimates (e.g. regression coefficient) AND variation (e.g. standard deviation) or associated estimates of uncertainty (e.g. confidence intervals)
☐	☒	For null hypothesis testing, the test statistic (e.g. F, t, r) with confidence intervals, effect sizes, degrees of freedom and P value noted<br>*Give P values as exact values whenever suitable.*
☒	☐	For Bayesian analysis, information on the choice of priors and Markov chain Monte Carlo settings
☒	☐	For hierarchical and complex designs, identification of the appropriate level for tests and full reporting of outcomes
☒	☐	Estimates of effect sizes (e.g. Cohen's d, Pearson's r), indicating how they were calculated

Our web collection on statistics for biologists contains articles on many of the points above.

Software and code

Policy information about availability of computer code

Data collection	*Provide a description of all commercial, open source and custom code used to collect the data in this study, specifying the version used OR state that no software was used.*
Data analysis	Agilent MassHunter Workstation Qualitative Analysis B.07.00 (Version: 7.0.7024.0), Excel 2013 (Version: 15.0.4569.1506), Agilent MassHunter Workstation Quantitative Analysis B.07.01 (Version: 7.1.524.0), Image J (R) (Version: 1.53e); GraphPad Prism (Version: 8.4.3)

For manuscripts utilizing custom algorithms or software that are central to the research but not yet described in published literature, software must be made available to editors/reviewers. We strongly encourage code deposition in a community repository (e.g. GitHub). See the Nature Research guidelines for submitting code & software for further information.

Data

Policy information about availability of data

All manuscripts must include a data availability statement. This statement should provide the following information, where applicable:

- Accession codes, unique identifiers, or web links for publicly available datasets
- A list of figures that have associated raw data
- A description of any restrictions on data availability

NCBI database (https://www.ncbi.nlm.nih.gov/)
UniProt (https://www.uniprot.org/)
Protein Data Bank PDB (https://www.rcsb.org/)
Modomics (http://genesilico.pl/modomics/)
Sequencing data: http://www.ebi.ac.uk/ena/browser/view/PRJEB41141
Source data for all main figures are provided with this paper. (link to source data)
More data that supports the findings of this study are available from the corresponding author upon reasonable request.

Field-specific reporting

Please select the one below that is the best fit for your research. If you are not sure, read the appropriate sections before making your selection.

- [x] Life sciences
- [ ] Behavioural & social sciences
- [ ] Ecological, evolutionary & environmental sciences

For a reference copy of the document with all sections, see nature.com/documents/nr-reporting-summary-flat.pdf

Life sciences study design

All studies must disclose on these points even when the disclosure is negative.

Sample size	The experiment was performed at least 3 times in independent biological replicates to allow statistical significance testing through student t-test. We assume an equal variance in both groups as chemical stress will most likely change the epitranscriptome in an ubiquitous manner. Furthermore, the potential change in mean (modification abundance) might be to higher or lower values (up-or downregulation) and thus we performed a two-sided t-test. A p-value below 0.05 is considered to be statistically significant.
Data exclusions	Due to overestimation of 2-methylguanosine in tRNA Phe after stable isotope dilution mass spectrometry, m2G was excluded from Figure 1 as suggested by a reviewer.
Replication	Triple Quadrupole experiments were performed at least 3 times and all attempts of replication were successful. Northern Blot (>3), RNA purification by SEC (>10), BioAnalyzer (>5) and other experiments showed high reproducibility over several experiments.
Randomization	As a major covariate, fluctuations in MS quantification efficiency was identified prior to analysis of samples. To exclude potential biases introduced by the order of sample measurements, stable isotope dilution mass spectrometry was performed. Furthermore, quality control samples werre added and addition "in-worklist" calibration measurements were performed to account for MS quantification bias. No fluctuations were observed throughout all experiments.
Blinding	We identified MS peak integration as the crucial step which introduces investigator bias. Thus, data quantification and peak integration was automatically performed by Agilent MassHunter Quantitative Software (e.g. peak integration) and data blinding was omitted.

Reporting for specific materials, systems and methods

We require information from authors about some types of materials, experimental systems and methods used in many studies. Here, indicate whether each material, system or method listed is relevant to your study. If you are not sure if a list item applies to your research, read the appropriate section before selecting a response.

Materials & experimental systems

n/a	Involved in the study	
x		Antibodies
	x	Eukaryotic cell lines
x		Palaeontology
x		Animals and other organisms
x		Human research participants
x		Clinical data

Methods

n/a	Involved in the study	
x		ChIP-seq
x		Flow cytometry
x		MRI-based neuroimaging

Eukaryotic cell lines

Policy information about cell lines

Cell line source(s)	HEK 293 (DSMZ), HeLa Acc-57 (DSMZ), HAP1 c631 (horizon discovery)
Authentication	The cell lines were authenticated by the vendors. HEK 293 and HeLa Acc-57 cells were authenticated by standard STR DNA typing (https://www.dsmz.de/collection/catalogue/human-and-animal-cell-lines/identity-control/authentication-of-cell-lines). HAP1 cells are clonally isolated and not authenticated as no reference sequence is available at ATCC.
Mycoplasma contamination	The HEK 293 cell line was tested negative for mycoplasma contamination. Other cell lines were not tested with commercial kits. However, tRNA modification analysis of all cell lines included 6-methyladenosine - a modification highly similar to 2-methyladenosine. 2-methyladenosine is only detectable in bacteria and mycobacteria. The absence of 2-methyladenosine signals in cell culture tRNA preparations is a clear indicator of absence of bacteria and mycoplasma.
Commonly misidentified lines (See ICLAC register)	No commonly misidentified cell lines were used in this study.
