# Supplementary material for: Cell culture NAIL-MS allows insight into human tRNA and rRNA modification dynamics in vivo
Source: Nat Commun. 2021 Jan 15;12:389. doi: 10.1038/s41467-020-20576-4 (PMC7810713; doi:10.1038/s41467-020-20576-4)
Supplement: Supplementary file 3 — Source Data [file 41467_2020_20576_MOESM3_ESM.zip › Overview of raw data for SI.docx]

**Raw Data for Supplement**

**Legend**

- No raw data available
- Find raw data in 🡪**specified file**

**Figures**

1. Northern Blot 🡪 **FigureS1 NorthernBlots**
2. Sequencing 🡪 Motorin lab
3. Biosynthesis & HRMS
4. HRMS
5. HRMS
6. HRMS
7. HRMS
8. HRMS
9. HRMS
10. HRMS
11. MRM signals, Increase of labeled nucleosides (data for Figure S12)
12. Increase of labeled nucleosides 🡪 **FigureS12 – appearance of new nucleosides**
13. HRMS
14. Micrographs of cells
15. BioAnalyzer of purified RNA species (already “uncropped”)
16. Validation, Influence of labeling 🡪 **Figure3b+c - Validation**
17. Phe maturation m5U & Psi 🡪 **Figure4 – tRNA Phe maturation**
18. tRNA maturation in detail 🡪 **Figure3e+5 – fwd rvs tRNA in detail**
19. 18S maturation in detail 🡪 **Figure5 – 18S rRNA**
20. LD50 MMS 🡪 **FigureS20 – LD50 MMS**
21. Phe transcription rate after MMS 🡪 **Figure6 – MMS Disc Phe**
22. Phe MMS discrimination 🡪 **Figure6 – MMS Disc Phe**

**Tables**

1. All tRNA Phe Quant mods 🡪 **(Figure1 – Phe Quant)**
2. Sequences of Cy3-Oligos
3. DMRM method
